# Josiah Bacon

**Published:** 1879-05

**Authors:** 


### EDITORIAL, ETC.

---

*The Death of Josiah Bacon*, Agent of the Rubber Company.

The following account is from the San Francisco *Chronicle*, of April 15, 1879, and shortly after its publication, Dr. Samuel B. Chalfant surrendered himself, and acknowledged that he had shot Mr. Bacon after a conversation between them, in which the latter had used some very insulting and irritating expressions.

We do not believe the murder was premeditated, but was the act of a sudden unfortunate impulse :

"The murder of Josiah Bacon, on Sunday morning, at the Baldwin Hotel, is one of the strangest on the criminal record of this city. A man, while preparing quietly in his room for church, hears a knock at his door, opens it, encounters a person who, with scarcely a word shoots him, and he falls dead. The assassin walks out of the hotel. No one is disturbed. The occupants of the adjoining rooms are breakfasting below. Only one person on the floor above hears the shot, scarcely louder than an echo but thinks nothing of it. The dead man lies on the floor of his room, the door being ajar, all the forenoon, the life and bustle of the great building going on around him. The situation suggests that in Hawthorne's 'House of the Seven Gables,' where Judge Clifford sits in an arm-chair in his room of the old house all night amid strange sounds and ghostly sights. Some one passing during the forenoon sees him lying there, but thinks it may be intoxication and does not interfere.

The Chambermaid who comes about noon, is the first who discovers the truth. There is no external hemorrhage, and it is at first thought that the man has died of heart disease. The body is taken up and carried to the Morgue, and there the ghastly wound is found in the abdomen. The first theory is that of suicide, and it is but gradually that people begin to think a murder has been committed. The details may be given more fully as follows: Mr. Bacon has been for twelve or fifteen years the representative of the Goodyear Vulcanite Company so far as concerned their relations to the dentists. Every dentist has had to pay the company $35 per annum, or $50 if the amount was not forthcoming on a certain day. The sum was no great tax on those who had any work at all, though a heavy burden to the members of the profession who are just beginning business. Having the law on his side, and not being a man of the gentlest manners, he frequently made enemies needlessly. Sometimes dentists would pretend that they had no business to escape the tax, and sometimes they would refuse payment entirely, and a lawsuit would result. The Courts have always decided against the dentists, and all who could afford it, however, have slowly come to accept the situation, and have made the yearly payment promptly. Mr. Bacon has had more or less trouble with the San Francisco dentists, and some of them who felt most bitterly have threatened to kick him out of their offices. Some would not notice him when they saw him. About the time of Mr. Bacon's arrival in the city ten days ago, one of them of whom he demanded a royalty, told him he had done no work for a year. A day or two afterward, being at a party, and the question of the Goodyear patent coming up, a lady told him she had some of his work in her mouth. He asked who made them, and she gave him the name of the dentist who had told him he had done no work for a year. Of course the agent vowed vengeance upon the recusant dentist. That he feared violence was evident from the fact that, being a small man, he took a stalwart companion with him wherever he feared trouble. His professional difficulties furnished the first clew to the police. The other traces of the crime were as follows: Last Thursday afternoon, a well-dressed and good looking man, who seemed to be 30 or 35 years of age came to the hotel, and after looking over the register, asked Mr. Marvin, the clerk, if Bacon was in. The reply was that he had gone to the country. On Friday evening he called again but Bacon had not returned. He asked for a personal description of him, which was given. It was about 9 o'clock when he came on Sunday morning. Bacon had been down, asked for his washing, which had not been sent home, and had gone to get shaved. The clerk thought that he had left the barber-shop and would be in his room. He so informed the stranger, who left the counter and walked toward the elevator. After that the visitor was not seen again. He seems to have knocked at the door, and Bacon, who had combed his hair and was adjusting his necktie preparatory to going to Dr. Stone's church, the Congregational Church in which he was brought up in Boston, turned and opened it. The bullet from the pistol, which was probably a Deringer, struck him below the navel, ranging downward. The autopsy is not finished, and it has not yet been found. He fell backwards, his head striking against the wall that divided his room from the corridor, and his feet toward the folding-doors, which opened into his bedroom. Some of his hair adhered to the wall.

The room is at the end of the hall of the third floor, and runs parallel with Powell Street. There is a window at the extreme end of the hall looking out on Ellis, and the door is the last on the left side. Nothing in the room was disturbed. Dr. Fox and family occupy the room on one side, and Mr. Gillan the room opposite, looking out on Ellis. They were all at breakfast. It was Dr. McAllister in the room who heard the faint report of the pistol. The body was taken to the Morgue about 1 o'clock. The description of the man who had inquired for Bacon was given to the police, who at once began to work up the affair diligently, under the special direction of Captains Lees and Stone. It was surmised at once that the deed had been done by an aggrieved dentist. There were other theories that the deceased had met his death by the hands of an enraged husband or lover, but they were not heeded. Inquiry about the dentists' offices showed that all of them were at work as usual except one, Dr. Samuel P. Chalfant, of the New York Dental Gallery, No. 19 Sixth Street. He could nowhere be found. His assistant, Mr. Richards, said that Chalfant left his office at 10 o'clock Sunday morning, promising to be back soon and keep an appointment he had made with a friend. Since then he has not been seen. Richards waited in the office all day, and when he left he closed the door, which had a spring lock, expecting to find his employer there when he came in the morning, and having no key was obliged to enter by the window. Upon investigation, it appeared that Chalfant had had a great deal of difficulty with Bacon, as representative of the Vulcanite Company. His diploma showed that he graduated at the Philadelphia Dental College in 1871. He practised his profession in Philadelphia, Wilmington, Del., and St. Louis, and in each of these places contested a suit brought by the company and invariably lost it. It would appear from statements made to the police, that he never paid the judgment against him, but before it could be enforced, sought new fields of labor. He therefore came to this city about four years ago. Bacon's business took him to nearly every city in the country. He came here in due course of affairs, stopping first at the Palace and then at the Baldwin. Finding Chalfant still using the patent and still refusing to pay the royalty, he brought suit against him in Justice Sawyer's Court on the 11th inst. On the examination he was cross-questioned by Bacon in a manner that galled him bitterly. He regarded himself as being unjustly pursued by a vindictive man representing a grasping corporation, and he terribly resented a feeling, perhaps, encouraged by Bacon's manner. As he used to say privately, if God had given a man a set of teeth and he had lost them, he thought that a dentist had a right to put them in without asking permission of, or making payment to anybody. The complainant in the case was represented by D. P. Belknap. The facts reluctantly extracted from him by the examination of B. Brown, were that he began practice at Wilmington, Del., in 1873. He remained there one year, and that he came from St. Louis to San Francisco. He had used the vulcanite all that time. In 1873 he made 150 plates for teeth ; in 1874, 200 ; in 1875, 200 ; 1876, 200; 1877, 200; 1878, 200, and so far in 1879 some 50 or 60. He also stated that he had been enjoined from using the material, and violated the injunction ; also, that Richards was not his partner. Examining his record previous to his professional life, the officers found that he had served during the war, having been Sergeant in Captain Samuel Cascadin's company D, Fifty-second Pennsylvania Volunteers, from 1861 to 1865, and that in the last year he joined the Veteran Corps.

Very thorough search was made by the police yesterday in every part of the city where it was thought Chalfant might be, but without the slightest trace of him. It was presumed that he had committed suicide, as his friends said he would never be taken alive. Every route out of the city was examined, including the Australian steamer which lay until midnight in the stream, but it did not appear that he had tried to flee the city. He is described by the police as a stout man, with black hair, dark brown mustache, very minute side whiskers, broad, square forehead, face broad and features regular, chin square and inclined to be double, nose slightly retrousse, and bright and sparkling eyes. His weight is about 160 pounds. He was dressed when last seen in a black business suit, round-topped hat, and gold watch and chain. He was highly esteemed by his friends as very companionable, warm in his nature and generous to a fault. His picture shows a firm and resolute character in every lineament. If he did the deed, it indicates dark, bitter, uncompromising qualities, of which his friends never dreamed. It seems strange that, if he planned the matter, he should have so publicly attempted its consummation ; that he should several times have gone to the office of the hotel to inquire for his man; that he should have asked his description ; that he should have shot him on Sunday morning, when everybody was in the hotel, instead of waylaying him outside. He could not hope to escape detection, and probably did not intend to survive his victim.

Josiah Bacon, was a brother of School Director J. S. Bacon, and has also a cousin in this city who is a lawyer. He was a small man, probably not more than five feet six inches in hight and weighing less than 150 pounds. A certain dogged perseverance in his character, while it made him bitter enemies, fitted him for the business in which he was engaged. He was born in Boston, and came to this city unknown to his parents when but 18 years of age. A few years afterwards he married a San Francisco lady, the wedding taking place at the house ot his cousin. His home since returning to the East has been chiefly in Boston. His wife is at present residing in Bridgewater. Inquiry among the dentists does not show that the better class of them object strongly to paying the $35 royalty. Most of them have paid it promptly, and only the less prosperous have objected, although none of them specially liked Bacon."

Since the above was prepared, a letter from San Francisco informs us that we were correct in supposing that the act of Dr. Chalfant was not premeditated, but was the result of a sudden impulse instigated by threatening language on the part of Mr. Bacon. The dentists of California, sympathizing with their unfortunate colleague, are endeavoring to raise a sum of money sufficient to meet the expenses of a proper defence, and the profession of the country are earnestly requested to contribute. Dr. S. S. White, of Philadelphia, has been suggested as a proper person to whom such contributions can be sent. We trust the profession will respond, as the sum of twenty thousand dollars is required.

